# Rediscovering empowerment with breastfeeding in an urban First Nation’s population

**DOI:** 10.1186/s12884-019-2631-x

**Published:** 2019-12-19

**Authors:** Deborah Schroeder, Pamala Larsen, Norma Jean Byrd

**Affiliations:** 1 Faculty of Kinesiology, University of Regina, 268 Leopold Crescent, Regina, SK Canada; 2 Faculty of Nursing, University of Regina, Regina, SK Canada; 3 Faculty of First Nations Health Studies, First Nations University of Canada, Regina, SK Canada; 4 Healthiest Babies Possible Program, Four Directions Community Health Centre, Regina, SK Canada; 5 Breastfeeding Committee of Saskatchewan, Saskatoon, SK Canada; 6 Saskatchewan Health Authority, 2011 Hamilton Street, Regina, SK Canada; 7 Faculty of Indigenous Health Studies, First Nations University of Canada, Regina, SK Canada; 8 AIDS Program South Saskatchewan, Regina, SK Canada

**Keywords:** Empowerment, Caregiving, Substance use, First nations, Supportive environments

## Abstract

**Background:**

An inner-city neighbourhood of Regina, Saskatchewan continues to have recurring issues of drug and alcohol use affecting parents’ caregiving opportunity. In relation to this, many children, mostly of First Nations descent, are raised in out-of-home care away from their families. With the promotion of breastfeeding, in a neighbourhood prenatal/postnatal support program, breastfeeding rates have doubled and mothers’ participation in their children’s care has increased. Recognition and promotion of cultural beliefs about breastfeeding is integral to raise community awareness of the practice. To bring additional support for breastfeeding, the empowerment effects observed were measured.

**Methods:**

Using a longitudinal study design, indicators of empowerment were assessed prenatally and again at two months postpartum. Indicators included self-esteem, caregiving activities, and drug and alcohol use. Outcomes of assessments were correlated to infant feeding practices and findings compared.

**Results:**

Findings supported a statistically significant improvement for empowerment scores when mothers breastfed. Mean scores for self-esteem increased from 2.87 to 3.57 (r = .90, *p* = <.001); for caregiving, scores increased from 2.60 to 3.16 (r = .91, *p* = <.001); and for drug and alcohol use, scores decreased from 59 to 9% (*p* = <.001).

**Conclusions:**

The study brings attention to the value of breastfeeding for caregiving in situations of addiction and limited resources. Practitioner reflexivity in regards to their support for breastfeeding is critical and includes openness to alternate breastfeeding situations and beliefs. The study found that a positive outlook on breastfeeding is the first step for a practitioner-client relationship that fosters confidence for marginalized populations.

**Significance statement:**

According to research, less breastfeeding occurs when mothers are marginalized. In turn, as marginalization increases, a mother’s self-esteem regarding her ability to adequately care for her child decreases. Healthcare professionals tend to be less likely to support a decision to breastfeed if there is concern about the mother’s resources and lifestyles. This research brings new attention to the importance of breastfeeding in disadvantaged situations related to an empowerment effect of breastfeeding for caregiving which includes cessation of drug and alcohol use. This effect has not previously been measured.

## Background

In Regina’s North Central neighbourhood, issues of addiction continue to jeopardize the parenting roles of First Nations mothers. Residents of this neighbourhood frequently encounter poverty, injection drug use (IDU) and domestic violence – all of which affect their caregiving ability. In one support program offered to residents of the neighbourhood, breastfeeding re-established the caregiving role of mothers.

In the 1980s, a young First Nations population moved to Regina, Canada, looking for a new life, away from their culture and its traditions [1]. At this time, however, Regina had a burgeoning drug market with a sex-trade industry and gang affiliations to support it. With little support for positive lifestyle choices, many of the youth succumbed to a lifestyle of drug and alcohol use. This lifestyle continues for many within the population today, and has a profound affect on their parenting ability. In 2010, a review of the province’s child welfare system identified that 80% of children in government care were of First Nations descent, a population that makes up only 10% of the province’s population [2]. A national survey of human immunodeficiency virus (HIV) cases further identified that the number of cases in North Central Regina were three times greater per capita than anywhere else in the country; 92% were associated with IDU and 69% were associated with First Nations ancestry [3].

Healthiest Babies Possible Program (HBPP), a prenatal/postpartum support program in North Central Regina provides support to, on average, half of the neighbourhood’s expectant mothers. The program, set up by First Nations women in the community, brought support to mothers with caregiving roles. An increased focus on breastfeeding became a part of the program when the Baby-Friendly Initiative (BFI), a program promoted by the health region, was introduced. The BFI has been a part of the World Health Organization health action to support breastfeeding world-wide, particularly in emergent situations. In HBPP, commitment to breastfeeding was further strengthened with the recognition of breastfeeding as a part of women’s life-giving role in First Nation’s beliefs, recognized to promote a caregiving relationship for the mother with her infant. These beliefs also link mothers to the cosmic realm for ancestral support in this relationship with breastfeeding [4].

HBPP participants experienced empowerment effects from breastfeeding, which in turn significantly enhanced perceived ability to care for their infants in other activities. As self-confidence for caregiving increased, drug and alcohol use stopped. Realizing the power of breastfeeding to increase an overall sense of caregiving-empowerment, HBPP staff and participants set out to measure this effect to further promote mothers’ breastfeeding opportunity, not always supported by healthcare providers.

### First nations breastfeeding practice

**Fig. 1:**
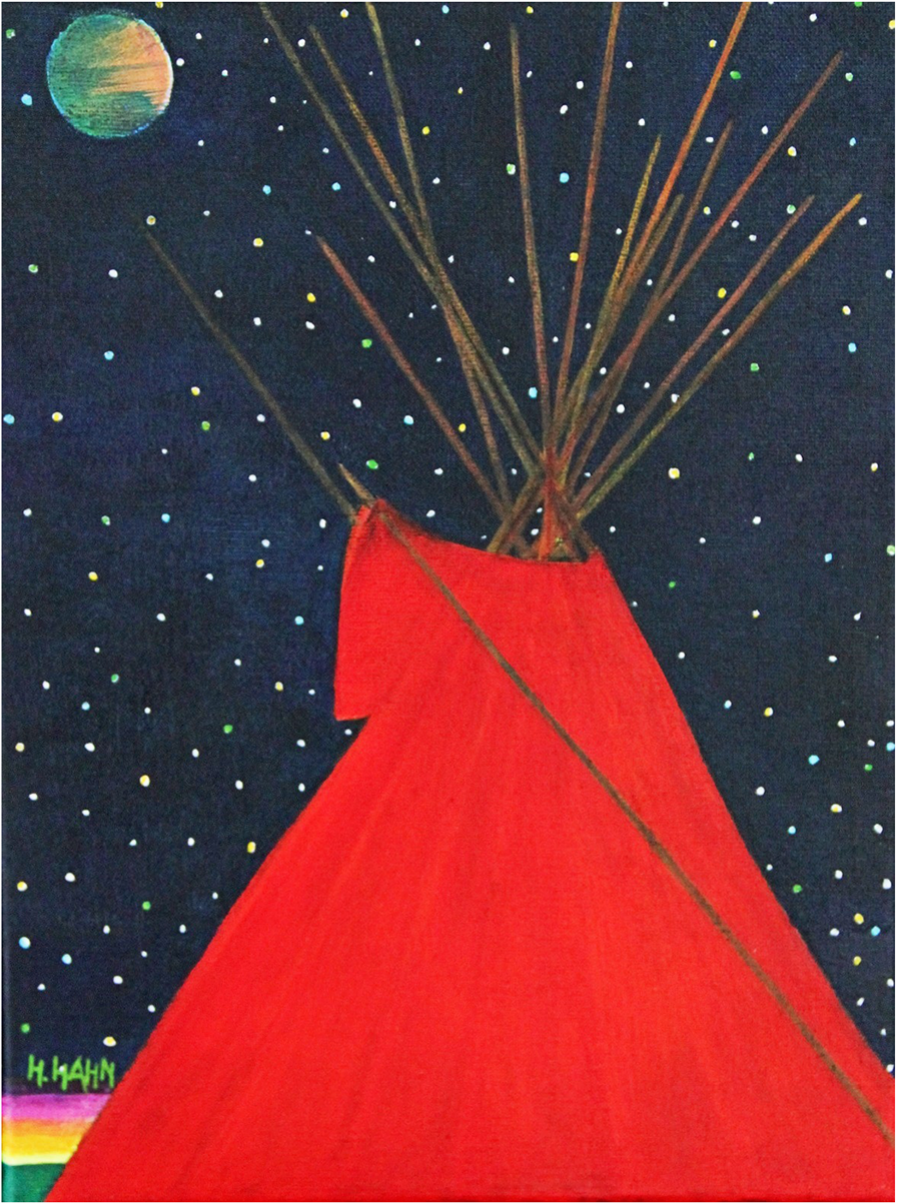
Written permission for the illustration “The Red Tipi” (Fig. 1) was provided by the artist Holle Hahn for use in this document, November 11, 2019

Caregiving was historically significant for women in First Nations tribes and included the kill of small game, planting of crops, care of children and childrearing that included teaching children traditional values [4–6]. The teepee, has been used as a symbol of mothers in this role. The poles are seen to be in the shape of a woman; and the buffalo skin like a shawl wrapped around her (see Fig. 1). The family is often shown inside the teepee further recognizing the mother’s protective role for her family [5]. Infants were kept in the constant watch of their mothers who carried them in a moss bag, secured to their backs. Keeping infants in this proximity encouraged the mother-baby relationship and promoted breast milk supplies. Fathers encouraged a high quality of breast milk by providing mothers with the best cuts of their “kill.” Other women in the tribe supported breastfeeding by assisting with the care of older children or breastfeeding an infant when the biological mother could not. This was done when Chief Crazy Horse, a memorable First Nations chief from the Lakota tribe, was orphaned as a young child. In addition to this support, older women, known as “Kohkoms,” assisted mothers with their knowledge of herbal remedies, such as the topical use of balsam to relieve engorged breasts [6, 7].

### Impact of Western society on first nations breastfeeding

Many First Nations people lost traditional cultural practices as a result of colonization and changes enforced by the Indian Act in 1876 [4, 8]. When land designation went to males instead of females, family roles were dismantled and the females’ leadership role for caregiving was undermined. First Nations food sources became food rations provided by colonizers, creating dependency by First Nations for their daily existence. Indian Agents, sent out by colonizers, enforced the use of milk powder for infants, thereby implying a mothers’ milk was substandard.

In a review of the Canadian National Database for Breastfeeding, a drop in Indian and Inuit populations’ breastfeeding was observed from 1960 to 1980 [9]. Early in the 1960s, breastfeeding rates were 69.4%, significantly higher than the 38% reported for the general population at that time. By 1980, breastfeeding rates had dropped to less than 60% in the Indian and Inuit population while the rates in the general population rose to 76%. A lower percentage of women breastfeeding within these populations continues today [10].

Traditional caregiving practices and support for breastfeeding came under new scrutiny when government workers were brought onto reserves to monitor mothers’ caregiving in the 1960s. Not fully understanding how the First Nations mothers’ caregiving had been devastated by colonization, First Nations women were portrayed by the workers “as demanding, filthy and prone to drunkenness.” [4] Government workers relocated many children far away from their homes, placing them in government foster care or residential schools in an action referred to as the *Big Scoop* [1]. It was this action that brought young people to Regina, seeking a sense of identity that fit with Western society and a life they believed to be better than what they would have remaining on their reserve. Ultimately, this move created devastation for the youth and their lives.

### Empowering mothers caregiving in disadvantaged circumstances

Research underlines the value of accomplishments with caregiving to create a positive self-worth by mothers dealing with disadvantaged circumstances, such as being adolescent, having low income or education, and marginalization in society [4, 11]. Positive affiliations with families and peers for caregiving are important to support caregiving practices such as breastfeeding. In one study, relocation of mothers from rural communities in India to urban Mumbai slums showed increased autonomy with caregiving despite continued partner abuse and isolation by their culture’s class system [12]. The change was linked to increased peer contacts and as well as observation of other mothers’ autonomy by social media. This led to increased responsibility for families’ healthcare, lifestyles and some income.

Locklin [13] noted an enhanced sense of self in the caregiving role for a small group of mothers from Hispanic descent with successful breastfeeding experiences. The mothers, who were multiparous, had attempted breastfeeding in the past, but discontinued prior to three weeks postpartum. With this experience, weekly support for breastfeeding was provided by peers who assisted the breastfeeding practise. Mothers became strong enthusiasts for breastfeeding, empowered by their breastfeeding success. Evaluations of the empowerment effect were limited, however, to self-reporting at a four-month postpartum interview.

A study conducted by Mossman, Heaman, Dennis, &. Morris [14] found adolescents who breastfed had increased self-worth, believing themselves to be valuable family members and having more confidence in social groups, enhanced by a sense of self following breastfeeding success. Evaluation of these indicators was at 28 days postpartum. First Nations mothers identified in the study were two times less likely to initiate breastfeeding as compared to other mothers, possibly related to a decreased sense of self with caregiving. Epstein [15], a research psychologist and breastfeeding activist, recognizes the value of a positive sense of self for mothers with breastfeeding. She credits the breastfeeding relationship between a mother and her infant to her ability to re-frame negative images of herself into positive ones. Caregiving accomplishments, as with breastfeeding, are recognized as significant to promote mothers’ caregiving roles, particularly when disadvantaged.

## Methods

While associations between positive self-images and caregiving functioning have been made in other Western research, a strong association between breastfeeding and its capacity to promote caregiving in high-risk situations has not yet been evaluated. The realization that breastfeeding empowered HBPP mothers to be stronger caregivers of their infants, identified by infant feeding, caregiving assessment and participation, emotional support, and follow up of infant health, created interest by the researcher to measure this effect. Commitment to measuring confidence with breastfeeding following the promotion of breastfeeding in the program was shared by mothers in HBPP. It was linked by some to their first real experience of unconditional love in a relationship.

The goal for the study was to empirically measure the correlation between breastfeeding and the mothers’ caregiving association while faced with challenges such as their own lack of role-modelling with this care, lack of resources and lifestyle risks. Both HBPP staff and participants recognized the value of such a measurement to support mothers’ breastfeeding opportunity, often challenged by healthcare providers and government workers. A longitudinal study design measuring caregiving indicators before and after breastfeeding, as well as when mothers did not breastfeed, was possible given mothers participation in HBPP until six months postpartum. The intent of the study was to establish breastfeeding as a catalyst for caregiving and a lifestyle free from drug and alcohol use.

Indicators of empowerment observed with breastfeeding included increased self-esteem, more participation in infant care and cessation of drug and alcohol use. Kabeer [16] recognizes such achievements to indicate an on-going empowerment process for caregiving when positive beliefs about caregiving ability are recognized by the mother. As achievements continue, these beliefs become deep-rooted, having a synergistic effect for confidence to assume additional activities with caregiving.

### Selection and description of population

All HBPP participants were invited to participate in the study over a six-month period. Most begin participation at around 16–20 weeks gestation. Expectant mothers more than 28 weeks gestation were excluded from participation due to the influence of elevated prolactin occurring by this time. Increased prolactin hormone levels and decreased activation of hypothalamic-pituitary-adrenal axis occurring with breastfeeding decreases the mother’s usual stress response potentiating her connection with the infant to enhance caregiving [17, 18]. Another variable promoting accurate measurement of empowerment indicators was the homogeneity of the group. Prenatal and postnatal support for breastfeeding was similar for study participants given their participation in HBPP. As well, participants came from the same neighbourhood and were dealing with similar disadvantages related to their First Nations ancestry and the undermining of cultural beliefs and practices by Western society.

Concern regarding mothers’ drug or alcohol use during infant care was monitored as a part of their HBPP participation. Interaction for this also prepared mothers for cessation of drug and alcohol use. Continued support by HBPP workers in the postpartum period allowed mothers’ lifestyle choices to be monitored to safe-guard infants from any possible change in lifestyle by mothers.

### Ethics and consent to participate

HBPP participants were included in all discussion of the study including empowerment indicators, study design, assessment tools and distribution of study findings. Study participants signed consent forms to participate in the study; however, consent did not include personal identification of information they shared. All study participants were over the age of 16 years. Canadian Indigenous Health Research governance recommends that researchers and communities should work collaboratively for research in a community-based process. This interaction is recognized to increase acceptance of research findings by the population and the relevance of recommendations for the population’s health [19]. OCAP principles (Ownership, Control, Access and Possession) further regulate research of First Nations populations in Canada, ensuring that a population has full input regarding the study, how it will be carried out and how the findings will be shared [19, 20]. The study was fully approved by the Saskatchewan Health Authority Research Ethics Board. On-going updates regarding study decisions and progress were provided to this board.

### Procedure and instruments

Expectant mothers were informed about the study at their HBPP placement and again at a prenatal health assessment carried out by the program’s registered nurse at around 25-weeks gestation. Consent was reviewed with the expectant mothers at the 25 week assessment and their consent for participation in the study was received at this time. The first interview for the study was completed following participant consent for participation. Mothers were invited to the second assessment at this time, which would be carried out at two months postpartum. A 20-dollar honorarium was provided following the second assessment. A celebration event was held at the conclusion of the study to acknowledge the contribution of the program participants to the study and to receive their feedback regarding study findings.

Previously established assessment tools were used to collect information regarding empowerment indicators. Tools were pretested with four program participants to obtain their feedback regarding the tools’ ability to assess empowerment indicators. Their feedback recommended that opportunity be given for additional participant comments at the end of the interviews for feedback regarding participant experience with the empowerment indicators. This reporting is suggested by other researchers as a means to enhance content validity with the use of closed-ended tools for data collection [21].

**A**ssessment of participants’ demographic information, including caregiving experiences, was collected using HBPP tools in the first interview. The tools were developed and tested by Barrington Researchers to evaluate program participants in Canadian Prenatal Nutrition Programs [22]. An intake questionnaire, developed by Addiction Services in the health region, was used to assess study participants’ drug and alcohol use. Both employ closed-ended questions. This assessment, however, was not a part of any program evaluation.

Rosenberg’s Self-Esteem Scale (RSES) measured self-esteem [23]. Respondents assessed ten self-esteem statements on a four-point scale ranging from “strongly agree” to “strongly disagree” with a higher score indicating higher self-esteem. The RSES has a coefficient of 73% for reproducibility, 72% for individual’s scalability and an internal consistency coefficient of .84 to .87.

Fawcett’s Inventory of Functional Status after Childbirth (IFSAC) assesses caregiving behaviors characteristic of optimum caregiving function [24, 25]. The IFSAC was used to collect data about caregiving activity by a study participant before and after giving birth. The tool contains five sub-scales measuring a mother’s caregiving functioning: family care, self-care, household activities, social and community activities, and education/occupation. Respondents rate a total of 36 items using a four-point scale. Higher scores indicate better caregiving ability. Content validity measured by the researcher for this tool was established as 96%.

Data was entered into an EpiInfo data base and analyzed using Version 16 of the Statistical Package for Social Sciences. Indicator measurements using instrument scores were reported. The T-test and Chi square analysis were used to evaluate sub-group differences. Correlation analysis examined the extent of the relationships between possible factors influencing a mother’s decision to breastfeed.

## Results

Study participation was maintained in conjunction with mothers’ participation in HBPP. Of the 53 mothers who agreed to participate in the study within a six-month span of invitation, 51 or 96%, completed both assessments (see Table 1). The two mothers who did not finish the last interview had moved to another location and could not be contacted for the final interview. However, only the data of those completing both assessments was used for evaluation. In the study group, 40 mothers chose to initiate breastfeeding but only 32 continued to breastfeed until the two-month interview (see Table 1). Eleven chose not to breastfeed for infant feeding. Two mothers identified as HIV positive and were not supported to breastfeed due to the risk of transmitting HIV to their infants through the breastmilk.

**Table 1:** Comparison of demographic characteristics to infant-feeding outcomes

	No	Attempted	Continued
	Breastfeeding	Breastfeeding	Breastfeeding
Demographic			
Characteristics			
(Sample *n* = 51)	(*n* = 11)	< 6 weeks (*n* = 8)	(*n* = 32)
Cultural Background			
First Nations (88%)	9 (82%)	7(88%)	28(88%)
Non-First Nations	2(18%)	1(13%)	4(13%)
Age in years			
< 18	1(9%)	1(13%)	1(3%)
18–26	5(45%)	5(63%)	23(72%)
> 26	5(45%)	2(25%)	8(25%)
Education			
< year 10	7(64%)	3 (38%)	9 (28%)
year 10–12	3(27%)	5(63%)	16 (50%)
> year 12	1(9%)	0(0%)	7(22%)
Relationship Status			
in a relationship	9(82%)	7 (88%)	19(59%)
single	2(18%)	1(13%)	13(41%)
Parity			
Primiparous	2(18%)	6(75%)	8(25%)
Multiparous	9(82%)	2(25%)	24(75%)
Breastfeeding experience			
None	9(82%)	8(100%)	19(59%)
Previous	2(18%)	0(0%)	13 (41%)
1st Doctor Visit			
< 3 month	6(55%)	4(50%)	21(66%)
> 3 month	5 (45%)	4(50%)	11 (34%)
Program Participation			
Every 1–2 week	4 (36%)	5 (63%)	22 (69%)
Once a month or less	7 (64%)	3 (37%)	10 (31%)
Care-provider			
Primary	6(54%)	8(100%)	28 (87%)
Non-Primary	5(46%)	0(0%)	4(13%)

### Indices of caregiving empowerment

**Fig. 2:**
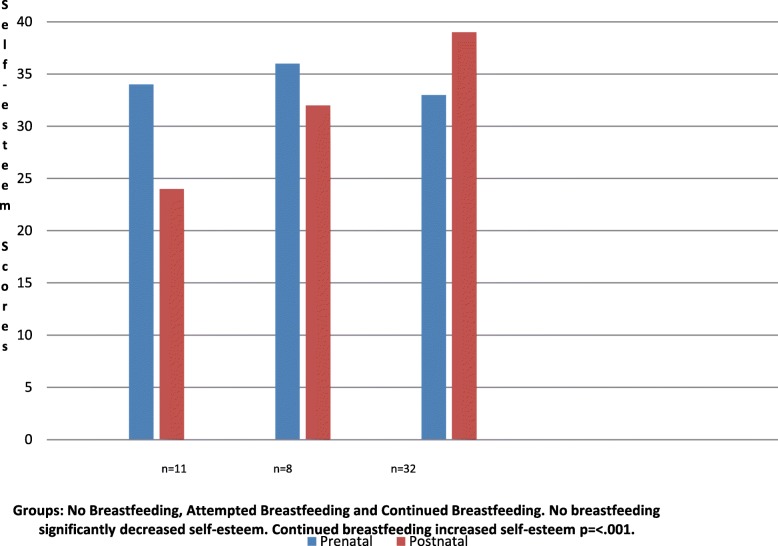
Means comparisons for self-esteem before and after infant-feeding outcomes

Indices of caregiving empowerment were measured and compared to the mother’s chosen method of infant feeding. A change in self-esteem from pregnancy to postpartum was significantly correlated to a mother’s infant feeding practice (see Fig. 2). The mean scores for mothers who continued to breastfeed at two months postpartum increased significantly from 2.87(33/44) prenatally to 3.57(39/44) postpartum (r = .90, *p* = <.001). For the mothers who did not breastfeed, there was a decrease in the mean self–esteem score from 3.05 (34/44) prepartum to 2.18 (24/44) postpartum (r = .90, *p* = <.001). The findings for mothers who did not breastfeed have limited value given the small sample size. However, other research of larger study groups established a decrease in confidence with caregiving when mothers did not breastfeed in larger study groups. These mothers were often identified to be adolescent, having limited resources or marginalized in some way [13–15, 26].

**Fig. 3:**
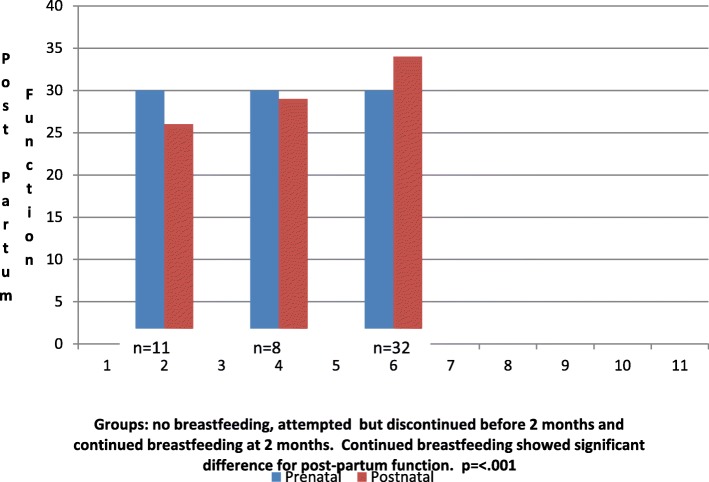
Means comparisons for care giving function before and after infant-feeding outcomes

**Fig. 4:**
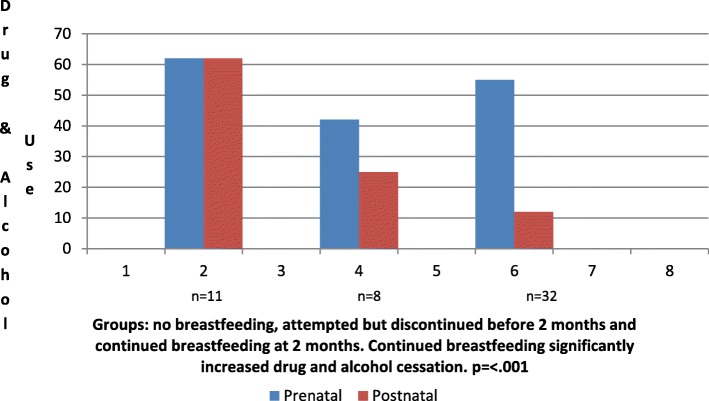
Percentage comparisons for drug and alcohol use before and after and infant-feeding outcomes

Significant change in caregiving function was also correlated to infant feeding choices (see Fig. 3). The average caregiving function scores of mothers who did not breastfeed decreased from 2.66 (31) prepartum to 2.26 (27) postpartum (*r* = .93, *p* = <.001). However, for mothers who continued to breastfeed at two months postpartum, the mean caregiving function score increased to 3.16 (35) postpartum (*r* = .91, *p* = 0.001) from 2.60 (31) prepartum. Lastly, the most significant change observed with breastfeeding was related to drug and alcohol use. For mothers who breastfed, there was a decrease from 59% (19/32) to 9% (3/32) (*p* = <.001) for drug and alcohol use, a dramatic difference compared to non-breastfeeding mothers, where there was no change in scores for drug and alcohol use (6/11) (see Fig. 4). During the second interview, several participants identified breastfeeding as becoming a “new addiction” and admitted returning to breastfeeding after weaning, afraid of losing the sobriety they had gained with breastfeeding.

### Correlations to breastfeeding decisions

Selected determinants of health, discussed in the “Toronto Charter for a Healthy Canada” (a national guide for health promotion) [27], were measured to identify their relationship to breastfeeding outcomes. Specific determinants consisted of social support networks, support of family and friends, and formal education. The staff’s experience with HBPP clients led to measurements of age, previous breastfeeding practice, and the presence of a partner as possible determinants affecting a mother’s breastfeeding choice.

Support from family and friends, in addition to program support, was found to be important for promoting breastfeeding (see Table 1). This was consistent with research findings which explored the value of this support to promote breastfeeding in disadvantaged situations [13–15, 21]. For mothers who initiated breastfeeding, 27 out of 40 (68%) attended HBPP programming every one to two weeks. Mothers were 88% more likely to breastfeed (35 out of 40) when support from family and friends was average or above average. Among the eleven that did not breastfeed, nine rated family and friend support as average or less (82%); only four attended programming more often than once a month.

Somewhat surprisingly, having a partner reduced the practice of breastfeeding (see Table 1). Of the mothers who continued to breastfeed at two months postpartum, only 19 of the 32 participants (59%) were with their partners as compared to 16 of the 19 (84%) mothers who were no longer breastfeeding. Overall, the mothers who were breastfeeding reported less contact with their partners due to partner drug and alcohol use. These women recognized that such a relationship could jeopardize their ability to continue parenting.

Breastfeeding initiation was most likely to occur when mothers were able to maintain primary caregiving responsibility of the infants (37/42 or 88%) (see Table 1). Nine study participants lost their infants to government foster care following delivery. All were multiparous and lost their infants because of past drug and alcohol use; none had ever breastfed. Through the assistance of HBPP staff, four maintained the use of their own milk for infant feeding by providing expressed breast milk to the foster parent. Two of these four mothers were reunited with their infants prior to the two-month postpartum interview.

Demographic information and postnatal experience provided insight for program support (see Table 1). Younger mothers, aged 18–26 years (28/33 or 85%), were more likely to initiate breastfeeding than those who were more than 26 years old, making them an especially important group to target for infant feeding information prenatally. Primiparous mothers were more likely than multiparous mothers to initiate breastfeeding; however, six of the eight mothers who discontinued breastfeeding were primiparious. This result suggests breastfeeding support in the postpartum continues to be important for this group of mothers. The majority of mothers who chose not to breastfeed were in the oldest age category, 26 years or older (5/11 or 45%), with most never having breastfed before (9/11 or 82%); they were also less likely to attend HBPP programming. Further examination of this situation is needed to understand how this group of mothers can be engaged to initiate breastfeeding.

## Discussion

The study underlines the importance of promoting caregiving practices, such as breastfeeding, that empower mothers’ caregiving association. This is true even when issues of drugs and alcohol are faced. Not supporting them with this choice can permanently jeopardize caregiving associations, as was seen in North Central Regina. Using the punitive approach of placing children in government care to promote caregiving association has only led to on-going issues of substance use, violence and loss of caregiving roles in the population. This research has found that re-establishing cultural identity with caregiving through breastfeeding has proven to be a more effective way to promote mothers’ caregiving association.

While diligence was asserted to promote the strongest evaluation possible with this population and their infant feeding, conclusions are limited to the mothers of this group. However, attention to the need for cultural perspectives in health promotion is identified in other reseach [27]. This type of promotion has led to members of the population experiencing an increased sense of self due to the realization that the strength of their beliefs can promote health and function [28–30]. Research guided by a community-based research approach is recognized to increase the population’s perspective regarding health practices and to better inform healthcare workers for their support of these practices [31].

For HBPP participants who chose to breastfeed, it was observed that their commitment to breastfeeding rights increased. This is consistent with Kabeer’s [16] recognition of an empowerment process with caregiving when achievements occur. Other research has linked the prohibition of showing emotions, particularly in the First Nation’s culture, to increased grief reactions, which is suggested to be a part of the healing process [32]. Several study participants challenged hospital, school and mall managers when their breastfeeding was not supported in these locations. In one situation, a meeting with the provincial government was set up following an insult experienced by one mother reprimanded for breastfeeding in the mall. This resulted in a breastfeeding-friendly statement coming from the ministry. These interactions further enhanced attachment to breastfeeding and recognition of its value for the infant and their own caregiving association. It is wondered if the pursuit by some participants later on of careers including nursing, addiction support work and paramedics could be linked to a positive breastfeeding experience.

Long-term benefits observed with breastfeeding for maternal health are relevant for this population given their health risks for diabetes and cancers. In a prospective Danish cohort study, an inverse association was observed for breastfeeding duration and weight retention up to 18 months correlating with anthropometric measures seven years after delivery [33]. For mothers who already have symptoms of gestational diabetes mellitus, breastfeeding has a protective effect against the development of type-2 diabetes [33].

In a large systematic review including more than 9000 abstracts and 29 systematic reviews and covering 400 individual studies, the effect of breastfeeding for long-term health of mothers and infants was investigated [34]. A history of breastfeeding was associated with reduced risks for type-2 diabetes, breast and ovarian cancer. Early cessation, or not breastfeeding at all, was associated with a higher risk of postpartum depression.

Attention has also come to the omission of breastmilk for use with infant feeding in the consideration of gross domestic product. In Norway, it is estimated that the market value of breastmilk, lost to formula and baby food sales totals $70 billion [35]. It is also estimated that health care costs incurred for premature deaths in United States, associated with low breastfeeding, is $4.38–24.68 billion [36]. Similarly, morbidity costs for diseases directly related to low breastfeeding are $733.77; indirect costs of disease are estimated to be another $126.1 million [36]. Likewise, in the United Kingdom, the costs for healthcare during 2009–2010 to treat illnesses associated with suboptimal breastfeeding was 957 million pounds for mothers and 89 million pounds for children [37].

Infant benefits directly from breastfeeding are linked to protection from infections and biological signals for promoting cellular growth and differentiation [38]. For example, living in crowded housing puts children at risk for respiratory infections in disadvantaged housing situations. Breastfeeding reduces the severity of respiratory problems particularly in the first 27 weeks of life. Recognizing this benefit, the American College of Obstetrics and Gynecology recommends breastfeeding infants exclusively until six months, followed by continued breastfeeding with complementary foods for one year or longer [38].

## Conclusion

The loss of traditional roles and functions as a result of colonization has contributed to issues of violence, abuse and dysfunction within many First Nations families. Children raised outside of their biological homes have been deprived of cultural knowledge relevant for roles and functions, as well as the sense of self with these. Support to re-establish this identity through experiences such as caregiving is needed for confidence with these functions.

Given the importance of breastfeeding for both the mother and baby’s physical and emotional health, American College of Obstetrics and Gynecology asks for a multidisciplinary approach involving practitioners, family members and child care providers to support breastfeeding in disadvantaged situations. Breastfeeding is recognized as a low-cost intervention which prevents cardiovascular disease, obesity and diabetes in high-risk women along with respiratory illness for the infant [39]. Initiatives like the BFI have an important role to play in assisting healthcare providers with reflection and reconsideration of their stance on relevant health issues. This could lead to more open-minded recommendations and approaches toward disadvantaged population members receiving their care.

The contribution of breastfeeding toward empowering a positive self-perception for mothers in the First Nations population cannot be undervalued. Breastfeeding is a powerful mechanism to enhance caregiving identity for all mothers. Additional research on the empowerment effect of breastfeeding in other populations is still needed to further support breastfeeding empowerment. The collection of breastfeeding experiences by traditional knowledge keepers is underway in the Regina population to further support identity with breastfeeding. Increased opportunities for mothers to breastfeed, along with collection of cultural knowledge for breastfeeding beliefs, offers a return to pride in caregiving identity and a sense of self once experienced by this population with caregiving.

## Data Availability

Availability of data and materials is available upon request from the primary author.

## Abbreviations

BFI: Baby-Friendly Initiative
HBPP: Healthiest Babies Possible Program
HIV: human immunodeficiency virus
IDU: injection drug use
IFSAC: Fawcett’s Inventory of Functional Status after Childbirth
OCAP: Ownership, Control, Access and Possession principles
RSES: Rosenberg’s Self-Esteem Scale
